# Antecedents and Consequences of Outward Emotional Reactions in Table Tennis

**DOI:** 10.3389/fpsyg.2020.578159

**Published:** 2020-09-15

**Authors:** Julian Fritsch, Emily Finne, Darko Jekauc, Diana Zerdila, Anne-Marie Elbe, Antonis Hatzigeorgiadis

**Affiliations:** ^1^Institute of Sports and Sports Science, Karlsruhe Institute of Technology, Karlsruhe, Germany; ^2^School of Public Health, Bielefeld University, Bielefeld, Germany; ^3^Faculty of Physical Education and Sport Science, National and Kapodistrian University of Athens, Athens, Greece; ^4^Sport Faculty, Leipzig University, Leipzig, Germany; ^5^Department of Physical Education and Sport Science, University of Thessaly, Trikala, Greece

**Keywords:** emotion, importance, controllability, behavior, observation

## Abstract

The purpose of the present study was to shed light on the behavioral component of emotions by investigating antecedents and consequences of outward emotional reactions during table tennis competitions. With regards to the antecedents of outward emotional reactions, in line with appraisal theories, we considered the importance and the controllability of the situation as two important constructs. Fifteen table tennis matches, involving in total 21 players (7 females) with a mean age of 16.71 (*SD* = 0.70), were video recorded during the finals of the youth National Championship in Greece. Based on the footage, outward emotional reactions after every point were classified as neutral, positive, or negative. Situational factors in relation to the scoring system, bearing the importance and the controllability of the situation, were formed to assess antecedents of outward emotional reactions. To measure the consequences of outward emotional reactions, the impact on the outcome of the next point was assessed. Generalized linear models with a logit link were computed separately for positive outward emotional reactions after having won a point and negative outward emotional reactions after having lost a point. In general, the results show that while situational factors bearing the importance of the situation could predict positive and negative outward emotional reactions, the effects of situational factors bearing the controllability of the situation were less conclusive. In addition, the results also showed interactive effects between the two constructs for both positive and negative outward emotional reactions. With regard to the consequences of outward emotional reactions, negative and positive outward emotional reactions could not predict the outcome of the next point. To conclude, this study highlights the behavioral component of emotions as a viable alternative to enhance our understanding of the role of emotions in sport.

## Introduction

In sports like table tennis, players can react very differently, depending on whether they won or lost a point. In some situations, after having won a point, players may make a fist and give a shout of joy, whereas after losing a point, players might throw the racket and give a shout of frustration. In other situations, however, you cannot really tell from the players’ reaction whether the point was won or lost. This variety of emotional reactions during a match not only makes sport fascinating to watch, it also says a lot about the psychological challenges players face in sports competitions.

Emotions are an integral part of sports. This is highlighted by studies in general psychology that make use of the nature of sports competitions to study emotions (e.g., Fernández-Dols and Ruiz-Belda, 1995; Matsumoto and Willingham, 2006; Aviezer et al., 2012). Although the definition of emotions is highly debated (Lindquist et al., 2013), researchers agree that emotions consist of one’s individual subjective experience (e.g., feeling anxious), physiological processes (e.g., change in blood pressure), and behavioral component (e.g., facial expressions; Mauss and Robinson, 2009). Research aimed at understanding the role of emotions in sports has mostly utilized questionnaires (e.g., Jones et al., 2005), or interviews (Martinent and Ferrand, 2009). Due to ethical and practical reasons, these methods cannot be applied to investigate emotions in real time, i.e., *during* real sports competitions, and thus limit the ecological validity of retrospective or prospective study designs (Uphill et al., 2014). As an alternative to these methods, focusing primarily on the subjective experience of emotions or the *perception* of physiological processes, this study was based on the premise that emotions are also observable from the outside. The behavioral component of emotion is worth investigating as it is relatively understudied, but also as it addresses the interpersonal nature of emotions (Tamminen and Bennett, 2017). Specifically, in this study we focused on the investigation of antecedents and consequences of outward emotional reactions in table tennis.

The term *outward emotional reaction* refers to an impression, which is based on an individual’s behavior and provides information about his/her emotional state. This impression includes different body signals that indicate an emotional state. Examples of these are postures, gestures, facial expressions, and verbalizations (Mauss and Robinson, 2009). It is important to note that instead of focusing on discrete emotions such as joy or anger, outward emotional reactions generally distinguish between positive and negative reactions, which makes it possible to study a wide range of emotions (Ekkekakis, 2013). While negative outward emotional reactions indicate that something emotionally unpleasant has happened to the individual (e.g., losing a point), positive outward emotional reactions indicate that something emotionally pleasant has occurred to the individual (e.g., scoring a point). It is further important to note that outward emotional reactions do not always correspond to the subjective emotions experienced. On the one hand, players sometimes fake a higher emotional intensity than is actually experienced through outward emotional reactions (Sève et al., 2007). On the other hand, even intense emotions experienced are not always accompanied by outward emotional reactions (Fernández-Dols and Ruiz-Belda, 1995).

From a sport psychological perspective, two intriguing questions arise: (a) under which conditions are outward emotional reactions more likely to occur and (b) how do these subsequently affect sports performance? In other words, it appears fruitful to focus on the antecedents and consequences of outward emotional reactions in sport competitions (Hanin, 2007). Expanding metacognitive knowledge about these two aspects could help players to regulate their behavior and thoughts more effectively and could thus also improve their emotion regulation skills (MacIntyre et al., 2014). Understanding the conditions under which players tend to react emotionally and identifying how these reactions affect sports performance can help us understand the underlying psychological processes. In addition, such knowledge could be used to develop strategies that channel emotions in a performance-enhancing way, both proactively and reactively (Uphill and Jones, 2007).

In sports like table tennis or tennis, winning or losing a point are the proximate events that trigger outward emotional reactions. Outward emotional reactions, however, are not randomly distributed throughout a match. In line with theoretical appraisal emotion approaches (Lazarus, 1991; Scherer, 2013), the importance of the situation and the controllability of the situation appear to be two relevant constructs that can either increase or decrease the likelihood of outward emotional reactions. These constructs do not themselves elicit outward emotional reactions, however, they may regulate the occurrence of outward emotional reactions following a potentially emotion-eliciting stimulus (i.e., winning or losing a point in table tennis). Because emotions are the result of a person-environment transaction (Lazarus, 1991), it is important to understand the subjective appraisal processes (e.g., Uphill and Jones, 2007). However, studies in tennis and table tennis have showed that these appraisal processes are often related to situational factors, such as the scoring system (Sève et al., 2007; Lewis et al., 2017). Since such situational factors can be objectively classified in observational studies, they appear to be particularly useful for studying their relationship to outward emotional reactions (for a study in handball see Moesch et al., 2015).

The importance of the situation hugely depends on the potential consequences it can have. Situations with more significant consequences for the individual’s goal attainment are associated with stronger emotional responses (Lazarus, 1991; Scherer, 2013). The importance of the situation is appraised very rapidly after the occurrence of an either positive or negative stimulus, and directs more in-depth processing (Scherer, 2013). Moesch et al. (2015) showed, for example, that handball players were more likely to celebrate a goal openly (i.e., positive outward emotional reactions) in elimination matches than in group stage matches. A possible explanation for this is that a defeat in elimination matches is equivalent to an elimination from the competition, which makes the situation more significant. In contrast, a defeat during a group stage match might be potentially compensated by subsequent matches. Because table tennis matches are divided into sets and sets in turn are divided into points, the importance can also be leveled down to the set level (within the match) as well as the point level (within the set). While points/sets closer to the potential end of a set/match have a stronger impact on the outcome of the set/match, earlier points/sets in the set/match can be compensated more easily by future actions.

The controllability of a situation refers to a basic psychological need, as people generally prefer to have control over the potential outcomes of a situation (Ryan and Deci, 2000). In comparison to the importance of the situation, which must be identified beforehand, the controllability of a situation is appraised at a later stage (Scherer, 2013). In addition, the controllability of a situation seems to be more important for stimuli that are incongruent with the current goal (Scherer, 2013). Since positive emotions are viewed as benefiting one’s goals (Lazarus, 1991), situational factors associated with the controllability of a situation could in particular influence the occurrence of negative outward emotional reactions. In table tennis, the current score is one relevant situational factor in relation to emotions (Sève et al., 2007). In situations where the players are leading, the controllability of the situation is higher than in situations where the players are trailing. Hence, the current score can be an indicator of controllability. Table tennis sets generally have 11 points. Leading by 9 to 2 points in a set indicates a higher controllability than leading by 9 to 8 points. At the same time, being behind by 2 to 9 points is associated with a lower controllability of the situation than being behind by 8 to 9 points. Furthermore, the controllability of the situation can be influenced by preceding experiences (Bandura, 1997). Winning consecutive points can lead to a positive momentum that in turn is related to a higher controllability of the situation (Kimiecik and Jackson, 2002). In contrast, losing consecutive points can lead to a negative momentum and is thus related to a lower controllability of the situation.

Summarizing the above we argue that a higher importance of the situation generally increases the likelihood of both positive and negative outward emotional reactions. Furthermore, a higher controllability of the situation generally decreases the likelihood of negative outward emotional reactions. As these constructs do not function in isolation during a sports competition, it is important to consider the interactive effects of their associated situational factors. For instance, losing a point when the score is 9-7 is related to a higher level of importance of the situation, which thus increases the likelihood of negative outward emotional reactions (Lazarus, 1991; Scherer, 2013). However, at the same time, being in front (9-7) increases the controllability of the situation, and thus decreases the likelihood of negative outward emotional reactions. On the contrary, losing a point at the end of the set when you are behind (7-9) can accumulate effects of a higher importance/lower controllability of the situation, making negative outward emotional reactions very likely.

From a sport psychological perspective, it is not only interesting to understand why players show outward emotional reactions in some situations and not in others, but also how these in turn affect the subsequent sports performance. Enhancing the understanding of the relationship between emotions and sports performance can raise players’ awareness of the importance of applying appropriate emotion regulation strategies (Jones, 2003). A number of studies have investigated outward emotional reactions (e.g., over verbalizations and gestures) and have linked these to objective performance indicators (e.g., the subsequent point). Studies in tennis have yielded interesting yet inconsistent results. While two studies showed an association between negative outward emotional reactions and a reduced probability of winning the next point (Van Raalte et al., 1994; Zourbanos et al., 2015), another study could not replicate this finding (Van Raalte et al., 2000). Conversely, it was shown that positive outward emotional reactions either have only a very weak positive association with the outcome of the next point (Van Raalte et al., 2000; Zourbanos et al., 2015) or no association at all (Van Raalte et al., 1994).

In this article, we argue that investigating outward emotional reactions can contribute to our understanding of the role of emotions in sports. The purpose of this study was to study emotions in real time and in their natural context by investigating antecedents and consequences of outward emotional reactions. In particular, we examined situational factors associated with the importance of the situation and the controllability of the situation as two important constructs influencing emotional outcomes (Lazarus, 1991; Scherer, 2013). With regard to antecedents of outward emotional reactions, we hypothesized that situational factors bearing a higher importance of the situation (e.g., point at the end of the set) would increase the likelihood of both positive outward emotional reactions after winning a point as well as negative outward emotional reactions after losing a point. In line with the assumption that the controllability of a situation has a greater effect for goal incongruent stimuli (Scherer, 2013), we also hypothesized that situational factors bearing a higher controllability of the situation (e.g., leading) would reduce the likelihood of negative outward emotional reactions after losing a point. For positive outward emotional reactions after winning a point, we hypothesized that the controllability of the situation would not have an effect. In addition, we hypothesized that the increased probability of negative outward emotional reactions in highly important situations would be reduced when there was a higher controllability of the situation (e.g., leading at the end of the set). For positive outward emotional reactions, we hypothesized that there would be no interaction between the importance and the controllability of the situation. With regards to the consequences of outward emotional reactions, the subsequent point was taken as a performance indicator. Given the inconsistent results of previous studies (Van Raalte et al., 1994, 2000; Zourbanos et al., 2015), we did not have specific hypotheses regarding the impact of positive and negative outward emotional reactions on the likelihood of winning the next point.

## Materials and Methods

### Participants

Participants were approached before the finals of the Greek national youth championship. A total of 14 male and 7 female Greek junior table tennis players, aged 16 to 18 years (*M* = 16.71; *SD* = 0.70) and with an average of 7.29 (*SD* = 1.72) years of competition experience agreed to participate in the study. The highest level of competition participation was international for 12 players and national for 9 players. The players trained 3.71 days (*SD* = 1.48) on average per week.

### Procedure

The university’s ethics committee gave its approval for the study. Players who were 18 signed a consent form, whereas for younger players the consent was granted by their legal guardians. In order to avoid self-presentation biases, the players were told before the match that the recording was intended for motion analysis. After the tournament, they were informed about the actual objective of the study. A total of 15 matches were recorded by two cameras positioned diagonally behind the table, which recorded the movements of the players on the opposite side. Thus, the video footage showed the whole person, including postures, gestures, facial expressions, and verbalizations, as important signals for the emotional state (Mauss and Robinson, 2009). In addition, the video footage also contained the trajectories of the ball, which allowed to keep track of the score. The data collection involved eight group stage matches, one quarter-final, four semi-finals, and two finals. Some of the players took part in more than one match, and a total of 2014 points (per match: 51 to 117; *M* = 67.13; *SD* = 17.6) were observed.

### Measures

#### Outward Emotional Reactions

Outward emotional reactions were used as a dependent variable to investigate their antecedents, and as an independent variable to investigate their consequences. Based on the video footage, two coders independently classified the players’ reactions after each point into (1) a positive outward emotional reaction, (2) a negative outward emotional reaction, or a (3) neutral outward emotional reaction. The first coder was a researcher with expertise in emotion literature and the second one a former Greek professional table tennis player and currently a coach. The coding was based on different body signals such as postures, gestures, facial expressions, and verbalizations, indicating the players’ emotional state (Mauss and Robinson, 2009). Since research suggests that conclusions about an individual’s emotional state cannot be drawn from physical features alone, but rather depend on context (Kayyal et al., 2015), the outcome of the point was taken as relevant context information. The coders were instructed to code outward emotional reactions as positive when the players’ behavior indicated that something emotionally positive occurred, and as negative if the players’ behavior indicated that something emotionally negative happened. Considering that individuals are always in some kind of emotional state (Russell, 2009), if the coders could not tell from the behavior of the players that something emotionally positive or negative occurred, these outward emotional reactions were coded as neutral.

#### Antecedents of Outward Emotional Reactions

##### Importance of the situation

Three situational factors representing the importance of the situation were analyzed, namely the stage of competition, the number of remaining sets, and the number of remaining points.

###### Stage of competition

This variable was used to distinguish between matches that could be compensated by future matches, and matches that were decisive for remaining in the tournament, i.e., group stage and elimination matches. Thus, points in elimination matches would indicate a higher importance of the situation than points in group stage matches. This situational factor was coded as a binary variable, with elimination matches coded as ‘1’ and group stage matches coded as ‘0’.

###### Remaining sets

This variable was used to indicate the lowest number of sets that potentially had to be played until the end of a match. The lower the number of remaining sets, the more important the situation was. Thirteen of the 15 matches were played until one player had won three sets. Two matches (one final and one semi-final) were played until one player had won four sets. This situational factor was coded as a continuous variable, with a higher number indicating that potentially more sets had to be played until the end of the match.

###### Remaining points

This variable was used to indicate the lowest number of points that potentially had to be played until the end of a set. The lower the number of remaining points, the higher the importance of the situation was. The sets of all matches were played until one player had won 11 points, with at least two points difference between the players. Thus, with a score of 10-10, the set lasted at least up to 12 points (in case of a score of 11-11, up to at least 13 points, etc.). This situational factor was coded as a continuous variable, with a higher number indicating that potentially more points had to be played until the end of the set.

##### Controllability of the situation

Four situational factors were used to assess the controllability of the situation, namely leading in sets, leading in points, consecutive points won, and consecutive points lost. Furthermore, the two situational factors set difference and point difference were formed. However, it is important to note that these two variables only took into account the absolute set/point difference between the players, and could not distinguish whether the players were leading or not. Because this made an important difference in terms of controllability, two interaction effects (*set difference x leading in sets* and *point difference x leading in points*) were additionally analyzed.

###### Leading in sets

This variable was used to distinguish between situations where the players were leading in sets within a match and situations where the players were not leading (either trailing or tied). Trailing and tie were combined into one category because a tie implies a set difference of ‘0’. Treating a tie as a separate category would have therefore resembled the variable set difference (see below), which would have led to collinearity and exclusion of this category. Leading in sets would thus indicate a higher controllability of the situation. This situational factor was coded as a binary variable, with leading coded as ‘1’ and trailing/tie coded as ‘0’.

###### Leading in points

This variable was used to distinguish between situations where the players were leading in points within a set, and situations where the players were not leading (either trailing or tied). The reason for combining trailing and tie was the same as described for the variable lading in sets (i.e., a tie would have resembled a point difference of ‘0’). Equivalent to the situational factor leading in sets, leading in points would indicate a higher controllability of the situation. This situational factor was coded as a binary variable, with leading coded as ‘1’ and trailing/tie coded as ‘0’.

###### Set difference

This variable was used to indicate the difference between sets won by each player. Since this variable had only three gradations, we formed two binary variables. While the first binary variable contrasted situations where an absolute set difference between the players was 1 (e.g., 2-1) to where it was a tie, the second binary variable contrasted situations where the absolute set difference between the players was 2 (e.g., 2-0) to where it was a tie. In both variables, the set difference of 1 set (or 2 sets) was coded as ‘1’ and a tie was coded as ‘0’.

###### Interaction set difference x leading in sets

For the variable set difference, only the absolute difference was taken into account, but not in whose favor the set difference was. For this reason, we computed the interaction between the two situational factors set difference and leading in sets. Because the results, upon using two dummy variables for the variable set difference, did not converge, the interaction effect was based on the continuous variable set difference.

###### Point difference

This variable was used to indicate the absolute difference between points won by each player within a set. The range of this variable was from 0 (in case of a tie) to the maximum of 10 (in case of a score of 10-0 or 0-10). This situational factor was coded as a continuous variable, with a higher number indicating that the point difference between the players was higher.

###### Interaction point difference x leading in points

Similar to the set difference, the point difference only took the absolute difference into account. For this reason, we formed an interaction term including the two situational factors point difference and leading in points.

###### Consecutive points won

This variable was used to indicate the number of points won in a row before the assessed point. A higher number of consecutive points won would indicate a higher controllability of the situation. The range of this variable was from 0 (in case it was the start of the set or the last point was lost) to a maximum of 10 (in case the score was 10-0). This situational factor was coded as a continuous variable, with a higher number indicating that more points were consecutively won by the players.

###### Consecutive points lost

This variable was used to indicate the numer of points lost in a row before the assessed point. In contrast to consecutive points won, a higher number of consecutive points lost would indicate a lower controllability of the situation. The range of this variable was from 0 (in case it was the start of the set or the last point was won) to a maximum of 10 (in case the score was 0-10). This situational factor was coded as a continuous variable, with a higher number indicating that more points were consecutively lost by the players.

##### Interaction between importance of the situation and controllability of the situation

Finally, we investigated the two interactions between remaining sets and leadings in sets as well as remaining points and leading in points in order to assess the interaction between the importance and the controllability of the situation.

###### Interaction remaining sets x leading in sets

This interaction allowed us to investigate whether the impact of the remaining sets on outward emotional reactions depended on whether the players were leading or not.

###### Interaction remaining points x leading in points

This interaction made it possible to investigate whether the impact of remaining points on outward emotional reactions depended on whether the players were leading or not.

#### Consequences of Outward Emotional Reactions

##### Outcome of next point

Here, we investigated whether outward emotional reactions were linked to the performance in the subsequent point. We predicted the outcome of winning, with a won point coded as ‘1’ and a lost point as ‘0’.

### Statistical Analysis

#### Statistical Analysis for Antecedents of Outward Emotional Reactions

The coders identified no positive outward emotional reactions after losing a point and only five negative outward emotional reactions after winning a point. For this reason, we made two different computations. The first computation included only the won points and positive (vs. neutral) outward emotional reactions as binary outcome, and the second computation included only the lost points and negative (vs. neutral) outward emotional reactions as binary outcome. Since some of the players participated in more than one match and up to 117 points per match were observed for each player, outward emotional reactions and match characteristics were not independent of each other, but were nested within persons and within matches. In addition, we considered a temporal autocorrelation since consecutive outward emotional reactions closer in time expected to be more similar.

We used generalized linear models with a logit link (i.e., hierarchical logistic models) within the R package ‘lme4’ (Bates et al., 2015) to predict outward emotional reactions as binary outcomes. We modeled match and player as two independent random effects. To account for the possible temporal autocorrelation, we created lag variables for the outward emotional reactions. That is, the outward emotional reactions before the observed point (and thus the corresponding outward emotional reactions as a dependent variable) were coded as the variables ‘lag 1’, the outward emotional reactions before it as the variables ‘lag 2’, and so on. This took into account the likely autocorrelations in the outward emotional reactions, since the lme4 package does not have an option to include autocorrelation errors as a specific covariance structure. Specifically, we looked at the outward emotional reactions as well as the outcome of the point of up to five preceding points. For the outcome of the preceding points, the lag variables were coded as ‘1’ when won or as ‘0’ when lost. For the outward emotional reactions, negative ones were coded as ‘-1’, positive ones as ‘1’, and neutral ones as ‘0’. Due to technical problems with the camera or situations in which the players were outside of the camera view, outward emotional reactions could not be coded in 93 situations. These outward emotional reactions were treated as missing values, however, for the lag variables we coded them as ‘0’ (neutral) to avoid a loss of cases.

To avoid convergence problems of more complex models, continuous predictors were grand mean centered, and the maximum number of iterations was increased. No issues with multicollinearity could be identified when looking at the generalized variance inflation factors of our models. Separately, for both positive and negative outward emotional reactions, we first calculated odds ratios for the bivariate relationships between each individual situational factor and the subsequent outward emotional reaction, adjusted only for the lag variables. In the final model, the significant interaction terms were included and the effects for each situational factor were adjusted by the effects of the other situational factors. The intraclass coefficient (ICC) was calculated for the empty model.

#### Statistical Analysis for Consequences of Outward Emotional Reactions

In order to assess whether outward emotional reactions affect sports performance, we also composed two models. In the first model, we tested the effects of positive (vs. neutral) outward emotional reactions, and in the second model, we tested the effects of negative (vs. neutral) outward emotional reactions. In both cases, this was then linked to the outcome of the next point (won vs. lost). The first point of each set was not included in the analysis because there was no immediate preceding outward emotional reaction. Since outward emotional reactions could also influence performance over more than one point, lag variables were also explored. Again, we looked at the outward emotional reactions as well as the outcome of the point of up to five preceding points.

## Results

### Descriptive Analysis of Codes

Overall, 1921 outward emotional reactions were recorded. In 93 situations (4.6%), players’ outward emotional reactions could not be coded due to technical problems with the camera or situations in which the players were outside of the camera view. Of the remaining 1921 outward emotional reactions, the two coders agreed on 1781 of the ratings (92.71%). With regard to the 140 differently rated situations, only one of the coders identified a negative outward emotional reaction in 119 cases, and a positive outward emotional reaction in 21 cases, whereas the other coder rated all of the 140 outward emotional reactions as neutral. In the following analysis, these 140 outward emotional reactions were treated as neutral, so that only positive and negative outward emotional reactions were considered as such in the analysis if they were identified as such by both coders. In the analysis, 480 negative outward emotional reactions and 481 neutral outward emotional reactions were coded after having lost a point and 356 positive outward emotional reactions, 5 negative outward emotional reactions, and 599 neutral outward emotional reactions were coded after having won a point.

### Antecedents of Positive Outward Emotional Reactions After Winning a Point

For the prediction of positive outward emotional reactions, none of the lag variables for preceding outcomes of the point were significant and, therefore, no lagged effects for the outcome of the point were included in the models. Since the three preceding outward emotional reactions were significantly related to the current outward emotional reactions, all following models were adjusted for these lagged effects. A model including only these three lagged effects of outward emotional reactions had a deviance value of 843.34, compared to 882.45 for the intercept only (empty) model (*χ*^2^(3) = 39.11, *p* < 0.05). The intraclass correlation (ICC) for person was 0.294, and for match was 0.367. The results for the individual situational factors are presented in Table 1, including odds ratio for the bivariate relationships and odds ratio adjusted by all situational factors and significant interactions.

**TABLE 1:** Antecedents of positive outward emotional reactions.

**Situational factor**	**Unadj. OR (95% CI)**	**Adj. OR (95% CI)**
**Importance of the situation**		
Stage of competition	6.314* (1.134–35.133)	14.965* (1.882–118.988)
Remaining sets^a^	0.583* (0.470–0.724)	0.782 (0.480–1.275)
Remaining points^a^	0.946^§^ (0.893–1.002)	0.775* (0.713–0.843)
**Controllability of the situation**		
Leading in sets	3.009* (1.882–4.811)	1.439 (0.684–3.030)
Leading in points	1.056 (0.705–1.582)	1.049 (0.546–2.015)
Set difference (1 vs. 0)	2.026* (1.359–3.019)	1.045 (0.494–2.212)
Set difference (2 vs. 0)	2.434* (1.882–4.811)	0.504 (0.157–1.621)
Set difference x leading in sets^b^		1.520 (0.419–2.771)
Point difference^a^	0.753* (0.641–0.864)	0.503* (0.404–0.626)
Point difference x leading in points		1.334* (1.016–1.752)
Consecutive points won^a^	0.865 (0.715–1.046)	0.810^§^ (0.636–1.033)
Consecutive points lost^a^	0.872 (0.728–1.044)	0.872 (0.688–1.105)
**Interaction between importance of the situation and controllability of**		
**the situation**		
Remaining sets x leading in sets		0.295* (0.141–0.617)
Remaining points x leading in points^b^		0.960 (0.839–1.098)

*^§^*p* ≤ 0.10; ^∗^*p* ≤ 0.05; ^a^Grand mean centered; ^b^This interaction was not considered in the final model.*

#### Situational Factors for Importance of the Situation

For the bivariate relationships, the two situational factors stage of competition (*OR* = 6.314, *CI* = 1.134–35.133) and remaining sets (*OR* = 0.583, *CI* = 0.470–0.742) were significant predictors of positive outward emotional reactions. In addition, the situational factor remaining points approached significance (*OR* = 0.946, *CI* = 0.893–1.002). In the final model, the two situational factors stage of competition (*OR* = 14.695, *CI* = 1.882–118.988) and remaining points (*OR* = 0.775, *CI* = 0.713–0.843) were significant predictors of positive outward emotional reactions. Thus, after having controlled for all situational factors, the results showed that after winning a point, the players were more than fourteen times more likely to show positive outward emotional reactions in elimination matches than in group stage matches. However, there was a large confidence interval. Furthermore, the likelihood of positive outward emotional reactions decreased by about 22.5% for each point further away from the potential end of the set.

#### Situational Factors for Controllability of the Situation

For the bivariate relationships, the four situational factors, leading in sets (*OR* = 3.009, *CI* = 1.882–4.811), set difference of one set (*OR* = 2.026, *CI* = 1.359–3.019), set difference of two sets (*OR* = 2.434, *CI* = 1.882–4.811), and point difference (*OR* = 0.753, *CI* = 0.641–0.864), were significant predictors of outward emotional reactions. In the final model, only the situational factor point difference (*OR* = 0.503, *CI* = 0.404–0.626) was a significant predictor of positive outward emotional reactions. Thus, after controlling for all situational factors, after winning a point the chance of a positive outward emotional reaction decreased by about 49.7% when the point difference increased by one point. In addition, the interaction between the two situational factors point difference and leading in points was a significant predictor (*OR* = 1.334, *CI* = 1.016–1.752). When the players were leading, the OR of point difference was 0.671, while when the players were trailing or tied, the OR of point difference was reduced to 0.503. This means that the effect of point difference on positive outward emotional reactions was moderated by leading in points. The effect was stronger when the players were trailing or tied. Finally, the effect for the situational factor consecutive points won approached significance (*OR* = 0.810, *CI* = 0.636–1.033). This suggests that the chance of positive outward emotional reactions decreased by about 19% when the number of consecutive points won increased by one point.

#### Situational Factors for Interaction Between Importance/Controllability of the Situation

The interaction of the two situational factors, remaining sets and leading in sets, was a significant predictor of positive outward emotional reactions (*OR* = 0.295, *CI* = 0.141–0.617). Specifically, when the players were leading, the OR for remaining sets was 0.231 and when the players were trailing or tied, the OR for remaining sets was 0.782. Thus, the results indicate that in situations with more remaining sets the chance of positive outward emotional reactions was more reduced when the players were leading compared to when they were trailing or tied.

### Antecedents of Negative Outward Emotional Reactions After Losing a Point

For the prediction of negative outward emotional reactions, again, none of the lag variables for the preceding outcomes of the point showed a significant effect, but we found significant effects up to the third lag variable for the preceding outward emotional reactions. A model including lagged effects for three preceding outward emotional reactions had a deviance value of 1249.40, compared to 1258.30 for the intercept only (empty) model (*χ*^2^(3) = 8.983, *p* < 0.05). We therefore included three lag variables, as in the models for positive outward emotional reactions. The intraclass correlation (ICC) for person was 0.036 and for match 0.021 in the intercept only (empty) model. Due to the low number of five negative outward emotional reactions after winning a point, they were not considered in this analysis. The results for the individual situational factors are presented in Table 2, including odds ratio for the bivariate relationships and odds ratio adjusted by all situational factors and significant interactions.

**TABLE 2:** Antecedents of negative outward emotional reactions.

**Situational factors**	**Unadj. OR (95% CI)**	**Adj. OR (95% CI)**
**Importance of the situation**		
Stage of competition	0.853 (0.545–1.335)	0.809 (0.504–1.299)
Remaining sets^a^	0.737* (0.628–0.865)	0.720^§^ (0.496–1.045)
Remaining points^a^	0.965 (0.923–1.008)	0.913* (0.862–0.968)
**Controllability of the situation**		
Leading in sets	1.038 (0.742–1.452)	0.947 (0.590–1.520)
Leading in points	1.053 (0.777 –1.429)	1.158 (0.819–1.637)
Set difference (1 vs. 0)	1.502* (1.111–2.031)	1.100 (0.623–1.942)
Set difference (2 vs. 0)	1.918* (1.286–2.861)	1.319 (0.577–3.016)
Set difference x leading in sets^b^		0.658 (0.344–1.257)
Point difference^a^	0.936^§^ (0.868–1.009)	0.831* (0.747–0.925)
Point difference x leading in points^b^		0.938 (0.788–1.116)
Consecutive points won^a^	1.098 (0.963–1.251)	1.115 (0.956–1.300)
Consecutive points lost^a^	0.981 (0.867–1.110)	1.032 (0.893–1.193)
**Interaction between importance of the situation and controllability of**		
**the situation**		
Remaining sets x leading in sets		1.924* (1.117–3.313)
Remaining points x leading in points^b^		1.020 (0.924–1.127)

*^§^*p* ≤ 0.10; ^∗^*p* ≤ 0.05; ^*a*^Grand mean centered; ^*b*^This interaction was not considered in the final model.*

#### Situational Factors for Importance of the Situation

For the bivariate relationships, the situational factor remaining sets was a significant predictor (*OR* = 0.737, *CI* = 0.628–0.865). In the final model, the situational factor remaining points was significant (*OR* = 0.913, *CI* = 0.862–0.968) and the situational factor remaining sets approached significance (*OR* = 0.720, *CI* = 0.496–1.045). Thus, after having controlled for all situational factors, the chance of negative outward emotional reactions decreased by about 8.7% for each point further away from the potential end of the set, and by about 28% for each set further away from the potential end of the match.

#### Situational Factors for Controllability of the Situation

For the bivariate relationships, the two situational factors set difference of one set (*OR* = 1.502, *CI* = 1.111–2.031), and the set difference of two sets (*OR* = 1.918, *CI* = 1.286–2.861) had significant effects. Furthermore, the situational factor point difference approached significance (*OR* = 0.936, *CI* = 0.868–1.009). In the final model, only the situational factor point difference was significant (*OR* = 0.831, *CI* = 0.747–0.925). This result suggests, after having controlled for all situational factors, that the chance of negative outward emotional reactions decreased by about 16.9% when the point difference increased by one point.

#### Situational Factors for Interaction Between Importance/Controllability of the Situation

The interaction of the two situational factors, remaining sets and leading in sets, was a significant predictor for negative outward emotional reactions (*OR* = 1.924, *CI* = 1.117–3.313). Specifically, when the players were leading, the OR for remaining sets was 1.385 and when the players were trailing or tied, the OR for remaining sets was 0.720. Thus, the results showed that in situations with more remaining sets the chance of negative outward emotional reactions was increased when the players were leading. On the contrary, when the players were trailing or tied the chance of negative outward emotional reactions was decreased with more remaining sets.

### Consequences of Outward Emotional Reactions

Finally, we conducted analyses to assess whether positive or negative outward emotional reactions can significantly predict the outcome of the next point. A total of 1813 observed points were included in this analysis. Thus, points where the immediately preceding outward emotional reactions were missing were not considered. This included those points where preceding outward emotional reactions could not be coded due to technical problems (*n* = 93) as well as the first point of each set (*n* = 108). Neither the lag variables for preceding outcomes of the point nor outward emotional reactions were significant and therefore no lagged effects were included in the models. No significant effects were found for both positive outward emotional reactions (*OR* = 0.954, *CI* = 0.734–1.239) and negative outward emotional reactions (*OR* = 1.117, *CI* = 0.892–1.399). Thus, the results suggest that neither positive nor negative outward emotional reactions could predict the outcome of the following point.

## Discussion

This study aimed to examine antecedents and consequences of outward emotional reactions during competitive table tennis matches. The results showed positive outward emotional reactions were more likely in elimination matches. Both positive and negative outward emotional reactions were less likely when there were more remaining points until the potential end of the set and also when there was a higher point difference between the players. For positive outward emotional reactions, the effect of point difference was stronger in situations where the players were trailing or tied than in situations where the players were leading. In addition, with more remaining sets until the potential end of the set, the chance of positive outward emotional reactions was more reduced when the players were leading compared to when they were trailing or tied. For negative outward emotional reactions, with more remaining sets until the potential end of the match, they were more likely when the players were leading in sets, but less likely when they were trailing or tied in sets. Finally, we found that neither positive nor negative outward emotional reactions could predict the outcome of the next point.

### Antecedents of Outward Emotional Reactions

Situations that are appraised as relevant for the individual’s goal realization have a greater influence on emotional responses (Lazarus, 1991; Scherer, 2013). It is therefore consistent with our hypothesis that situational factors representing the importance of a situation increased the likelihood of both positive and negative outward emotional reactions. The controllability of the situation is particularly relevant in the emotion generation process for stimuli that are not congruent with the current goal (Scherer, 2013). Thus, we hypothesized that the controllability of the situation would reduce the likelihood of negative outward emotional reactions, but would not influence positive outward emotional reactions. However, in contrast to this hypothesis, the results show that, after having controlled for all situational factors, none of the situational factors representing the controllability of the situation could predict the likelihood of negative outward emotional reactions; some of them, however, could predict the likelihood of positive outward emotional reactions. Below, we will first discuss the findings of the individual situational factors concerning the importance of the situation, then the factors related to the controllability of the situation, and finally the interaction of factors related to the importance and controllability of the situation.

#### Importance of the Situation

In accordance with our hypothesis, the results show that positive outward emotional reactions were more likely in elimination matches than in group stage matches. These findings are in line with a study with handball players (Moesch et al., 2015). Since a defeat in an elimination match cannot be compensated for in future matches, the outcome of a won point is more important. This makes the situation more likely to elicit emotions (Scherer, 2013). One possible explanation for the finding that the stage of competition does not influence the likelihood of negative outward emotional reactions is the concept of loss aversion, indicating that the fear of losing is associated with stronger emotional reactions than the hope of a possible win (Tversky and Kahneman, 1991). Irrespective of the stage of competition, there is a general tendency to react emotionally after losing a point, which may also explain why overall more negative than positive outward emotional reactions were identified in this study.

A confounding effect was found for the situational factor remaining sets for positive outward emotional reactions. While the effect was significant in the bivariate model, its significant interaction with the situational factor leading in sets may explain why it was not significant in the final model (this interaction effect will be discussed later). The results were less conclusive for negative outward emotional reactions. Although the effect only approached significance, a comparison of the statistical parameters showed a stronger effect in the final model, indicating that negative outward emotional reactions are more likely in sets closer to the end of the match. The outcome of a set closer to the end of a match can be decisive for the final result of the match, thus making the situation more important than the outcome of a set at the beginning of a match. In relation to the situational factor remaining points, its effect was significant in the final model, but not in the model with bivariate relationships, for both positive and negative outward emotional reactions. One can speculate that other situational factors in the regression model suppressed irrelevant parts of the variance in the variable remaining points. In addition, that more conclusive effects were revealed at the point level compared to the set level, is consistent with the observation that players often “reset” their mind after the end of a set (Lewis et al., 2017).

#### Controllability of the Situation

Similar to the importance of the situation, we found some confounding effects for situational factors with regards to the controllability of the situation. More specifically, the situational factors set differences (for a set difference of both one or two sets) were significant in the model with the bivariate relationships, but not in the final model, for both positive and negative outward emotional reactions. The same was found for the situational factor leading in sets, but only for positive outward emotional reactions. Thus, we assume that the effect of these situational factors overlaps with the effect of other situational factors. In contrast to our hypothesis, after having controlled for all other situational factors, none of the situational factors representing the controllability of the situation showed an effect on negative outward emotional reactions. This is surprising as the controllability is regarded as an important factor in the emotion generation process, especially for stimuli that are opposing the current goals (Scherer, 2013). Since appraisal processes related to the controllability of the situation come into play at a later stage in the emotion generation process (Scherer, 2013), this indicates a stronger impact of emotion regulation strategies. Since negative emotions are more difficult to regulate than positive ones (Martinent et al., 2015), this again could underline the potential role of loss aversion, which suggests that the effects of negative events are less dependent on situational factors (Tversky and Kahneman, 1991).

The point difference was the only situational factor that predicted both positive and negative outward emotional reactions in the final model. The smaller the point difference between the players was, the more likely an outward emotional reaction occurred. This is interesting because the point difference differently represents the controllability of the situation, depending on whether the player is leading or not. It could be that, regardless of who is leading, the predictability of the situation is one important facet in the appraisal process in sports competitions (Thatcher and Day, 2008). Thus, in times of a tight point score it is difficult to predict who will win the set, which generally increases the emotionality of the situation. Contrary to our hypothesis, it could further be shown that the situational factor leading in points moderated the effect of the point difference on positive outward emotional reactions. In both situations, players were more inclined to show positive outward emotional reactions when there was a small point difference. However, in times of a small point difference, the likelihood was higher when the players were trailing or tied than when the players were leading. In line with the observation that leading in matches is often associated with feelings of confidence and relaxation (Lewis et al., 2017), this can make the situation less emotional and therefore reduces the likelihood of positive outward emotional reactions. This may also explain the trend that positive outward emotional reactions became more and more unlikely when players were winning points consecutively.

#### Interaction of Importance of the Situation and Controllability of the Situation

We further assessed the interaction between the importance of the situation and the controllability of the situation. For negative outward emotional reactions, the results suggest that players who were leading in sets were less likely to show negative outward emotional reactions when there were less remaining sets until the potential end of the match. This is in line with our hypothesis that the controllability of the situation can counteract the effects of the importance of the situation. If players were leading in the final phase of the match, this can give them a sense of confidence and control (Lewis et al., 2017), which in turn reduces the likelihood of negative outward emotional reactions. In contrast, if players are trailing or tied in the final phase of the match, the effect of the lower controllability of the situation and the higher importance of the situation can reinforce each other. This may account for the increased probability of negative outward emotional reactions in such situations found in our study. Contrary to our hypothesis, the findings also revealed an interaction effect for positive outward emotional reactions. More specifically, it was shown that positive outward emotional reactions were more likely to occur in the final phase of the match when they were leading. Disengagement processes may explain why this effect was not as strong in the final phase of the match when the players were trailing or tied (Gaudreau et al., 2005).

### Consequences of Outward Emotional Reactions

We further explored whether outward emotional reactions can influence sports performance. Our study design allowed us to use the next point as an objective performance indicator that closely follows the emotion (Uphill et al., 2014). The results indicate that neither positive nor negative outward emotional reactions have an impact on the outcome of the next point. Although other studies that focused on overt verbalizations and gestures in tennis reported debilitative effects of negative and facilitative effects of positive outward emotional reactions (e.g., Zourbanos et al., 2015), our results are in line with studies that did not report an effect on performance (e.g., Van Raalte et al., 2000). The inconsistency of these findings demonstrates that predicting sports performance in general is a difficult task (Nevill et al., 2008), which could be supported by considering additional situational and personal variables (e.g., Moesch et al., 2018). Since emotions can have a stronger effect on observers when they are identifiable from the outside, the study of outward emotional reactions seems particularly suitable for understanding the interpersonal consequences of emotions, an area that has received scant attention in sport psychology (Tamminen and Bennett, 2017). There are several laboratory studies within the context of sport that indicate how outward emotional reactions can influence the opponents’ cognitions, emotions, and behaviors (e.g., Furley and Schweizer, 2014; Furley et al., 2015); however, research in the field is lacking. Given the importance of increasing the ecological validity of studies that focus on the relationship between emotions and sports performance (Uphill et al., 2014), we are convinced that with the increased use of innovative research methods (e.g., automatic recognition of body signals; Hetland et al., 2018), the explicit focus on outward emotional reactions can contribute to further insights in this area.

### Implications for Practice

The present results have various implications for applied work, which are worth examining in future studies. Although our results did not indicate a direct influence of outward emotional reactions on performance, it can be argued that there are situations in which players would benefit from emotion regulation strategies that prevent the occurrence of outward emotional reactions (Lane et al., 2012). Since our study suggests that in certain situations there is an increased likelihood of both positive and negative outward emotional reactions, it seems promising to tailor the use of sport psychological techniques to psychological demands of such situations (Martinent et al., 2015). Here, reflexive self-talk interventions could raise awareness of the players’ organic self-talk in such situations, which would possibly help to address potential irrational performance beliefs and subsequent outward emotional reactions (Latinjak et al., 2019). Furthermore, relaxation strategies (e.g., systematic breathing; progressive muscle relaxation) can help to deal with increased arousal in situations that are associated with a higher likelihood of outward emotional reactions (Pineschi and Di Pietro, 2013).

### Limitations and Future Directions

One limitation of the current study is the exclusive use of observational data. Although this methodology made it possible to study emotions ‘online’ in a real sports competition, an emotion is typically defined as having a subjective experience and physiological processes in addition to the behavioral component (Mauss and Robinson, 2009). In particular, the subjective experience is often considered the most essential component that distinguishes an emotion from other psychological states (Scherer, 2009). Nevertheless, outward emotional reactions imply consequences, especially with regards to the interpersonal side of emotions (e.g., Furley et al., 2015), which can hardly be explained by the subjective experience. For this reason, it seems that especially the combination of the different emotion components can contribute to a better understanding of the role of emotions in sports. In line with emotion theories (Lazarus, 1991; Scherer, 2013), importance and controllability were considered as potential moderators of outward emotional reactions and contributed to our understanding of the antecedents of emotions in sports competitions. Nevertheless, it is worth noting that these were operationalized based on objective criteria. For a better understanding of their role, future research could potentially explore their relevance for emotions through subjective measures focusing on individuals’ appraisal processes (e.g., Sève et al., 2007; Lewis et al., 2017).

Although the statistical analysis took the multi-level structure of the data into account, we did not explicitly deal with inter-individual differences. In sports competitions, however, it can often be seen that players differ in their tendency to show outward emotional reactions. As research points to individual dispositions influencing appraisal processes (Scherer, 2009), inter-individual differences could be another interesting line of future research. Moreover, in the current study we contrasted situations where players were leading with situations where players were not leading (i.e., for statistical reasons described in the methods section, trailing and tie was combined into one category). Because trailing or tied situations are qualitatively different from each other, future studies could consider this distinction. In addition, we only focused on whether or not positive/negative outward emotional reactions occurred, but we did not consider their intensity. It seems plausible that specifically outward emotional reactions with a high intensity (e.g., players make a fist and give a loud shout) have an effect on the opponent. Similar to the emotion experience (Hanin, 2007), a research design that takes their intensity into consideration could thus be specifically relevant with regard to sports performance. Finally, advances in technology appear to offer fascinating perspectives for future lines of research (e.g., Hetland et al., 2018). The automatic coding of physical features signaling outward emotional reactions could allow to draw on huge amounts of data in real sports competitions and thus help us to understand the role of emotions in sports.

## Conclusion

In conclusion, the present study employed a novel methodological perspective to investigate the role of emotions in table tennis. The findings provide valuable preliminary evidence of how various situational factors related to the importance and controllability of the situation influence the likelihood of positive and negative outward emotional reactions. This knowledge is useful both from a theoretical as well as a practical perspective to understand why players can react so differently to similar situations, such as winning or losing a point. Furthermore, our study underlines the importance of considering all emotion components, which, with the progress of new technologies, can help to unveil new insights into the relationship between emotions and sports performance.

## Data Availability Statement

The raw data supporting the conclusions of this article will be made available by the authors, without undue reservation.

## Ethics Statement

The studies involving human participants were reviewed and approved by ethics committee of the University of Thessaly Department of Physical Education and Sport Sciences. Written informed consent to participate in this study was provided by the participants’ legal guardian/next of kin.

## Author Contributions

JF wrote the manuscript. JF, DJ, AH, and A-ME conceptualized the study. JF and DZ organized the data collection. EF conducted the statistical calculations. DJ, AH, A-ME, and EF helped to edit the manuscript. All authors approved the final version of the submitted manuscript.

## Conflict of Interest

The authors declare that the research was conducted in the absence of any commercial or financial relationships that could be construed as a potential conflict of interest.
